# Carbon reduction multidimensional ternary model for robotic 3D printing prefabricated construction across hotels star levels

**DOI:** 10.1186/s13021-026-00447-z

**Published:** 2026-05-22

**Authors:** Gangwei Cai, Xiaoting Guo, Jian Chen, Qian Wu, Yiru Pan, Yifan Yao, Ye Lu, Yuguang Sun, Feidong Lu, Weijun Gao

**Affiliations:** 1 Institute of Landscape Architecture, College of Agriculture and Biotechnology, Zhejiang University, Hangzhou, China; 2 Hangzhou International Urbanology Research Center & Zhejiang Urban Governance Studies Center, Hangzhou, China; 3 Department of Architecture, Graduate School of Engineering, The University of Tokyo, Tokyo, Japan; 4 College of Artificial Intelligence, Zhejiang University, Hangzhou, China; 5 School of Civil Engineering and Architecture, Zhejiang University of Science and Technology, Hangzhou, China; 6 Faculty of Environmental Engineering, University of Kitakyushu, Fukuoka, Japan; 7 The Architectural Design & Research Institute of Zhejiang University Co., Ltd., Hangzhou, China; 8 Center for Balance Architecture, Zhejiang University, Hangzhou, China; 9 Baoye Group Co., Ltd., Zhejiang, China; 10 College of Architecture and Urban Planning, Tongji University, Shanghai, China; 11 Powerchina Huadong Engineering Corporation Limited, Hangzhou, China; 12 Zhejiang Tongji Vocational College of Science and Technology, Zhejiang, China; 13 Tongji Architectural Design (Group) Co., Ltd., Shanghai, China; 14 Institut Teknologi Sepuluh Nopember, Kampus ITS Sukolilo Surabaya, Surabaya, Indonesia; 15 The Engineering Academy of Japan, Tokyo, Japan

**Keywords:** Carbon reduction, Prefabricated buildings, Robotic 3D printing, Spatial-temporal analysis, Ternary model, Conventional environment habitats, Extreme environment habitats, Multidimensional algorithm, Sustainable tourism, Sustainable Development Goals (SDGs)

## Abstract

This study evaluates the carbon emission reduction potential of industrialized construction technologies—specifically robotic 3D printing and prefabrication—in lower-star hotels in Hangzhou, China, from 2018 to 2023. A multidimensional ternary spatiotemporal model is developed to quantify the interactions among temporal, spatial, and hotel classification dimensions, enabling a systematic comparison between conventional construction (CC) and prefabricated construction (PC). The results show that prefabricated construction consistently reduces embodied carbon emissions across hotel categories, with the most significant reduction observed in 2-star and 3-star hotels, where carbon emissions can be reduced by approximately 20–35% compared to conventional methods. The findings further indicate that carbon reduction effectiveness varies by hotel classification and spatial distribution, with lower-star hotels benefiting more from standardized modular construction, while high-star hotels exhibit relatively lower reduction efficiency due to structural complexity. Spatiotemporal analysis also reveals heterogeneous decoupling patterns across districts, suggesting that carbon–economic relationships are strongly influenced by regional development conditions and construction intensity. This study advances the integration of carbon accounting and construction technology assessment by providing a quantitative and scalable framework for evaluating embodied carbon reduction in the hospitality sector. The findings offer robust evidence to support targeted low-carbon construction strategies and contribute to carbon–economic decoupling pathways in resource-constrained urban environments, aligning with the scope of carbon balance and management.

## Introduction

The increasing urgency to address global climate change has brought carbon reduction and sustainable development to the forefront of research and policy agendas, particularly in energy-intensive sectors such as construction and hospitality. The hotel industry, as a core component of tourism economies, is characterized by high energy consumption and significant carbon emissions, posing challenges for the achievement of Sustainable Development Goals (SDGs) [1], notably SDG 8.9 and SDG 12.5. While previous studies have examined low-carbon practices in construction and tourism, limited attention has been given to the integration of advanced construction technologies—such as robotic 3D printing-based prefabrication—within the context of hotel buildings, especially in developing countries and lower-star segments [2]. In this study, “low-star hotels” refer primarily to 2-star and 3-star establishments characterized by limited budgets, smaller building scales, and basic operational facilities, which often reflect both lower economic capacity and resource constraints.

Emerging construction methods, including robotic 3D printing prefabrication, offer promising pathways for reducing embodied carbon and improving resource efficiency. However, there remains a research gap regarding the effectiveness of these technologies in the hotel sector, particularly when evaluated using comprehensive frameworks that capture spatial, temporal, and service-level dimensions. Therefore, this study aims to develop and apply a multidimensional ternary spatiotemporal model to assess the carbon reduction potential of robotic 3D printing prefabricated construction across different hotel star levels in Hangzhou, China, from 2018 to 2023. Hangzhou was selected as the case study because it represents one of China’s most dynamic tourism and hospitality hubs, featuring a large concentration of small and medium-sized hotels alongside ambitious municipal sustainability goals. The city’s policy emphasis on digital construction and low-carbon urban development provides an ideal context for examining the potential of industrialized construction technologies in practice.

This work seeks to contribute to the literature by filling the gap in empirical, sector-specific decarbonization analysis and by providing actionable insights for policymakers and practitioners working toward sustainable hospitality and urban development. This approach is particularly relevant as developing countries often face barriers such as limited capital, scarce resources, and harsh climates, which hinder the implementation of conventional carbon reduction strategies. Robotic 3D printing prefabricated construction not only overcomes these constraints through efficient resource utilization and adaptability but also delivers measurable environmental and economic gains. By targeting the retrofit of low-star hotels, this strategy aligns with global sustainability initiatives and provides a scalable, practical pathway for sustainable development in challenging contexts, thereby supporting both climate action and local economic resilience in line with the SDGs.

## Literature review

The Sustainable Development Goals (SDGs) and technological innovation have gained attention in low-carbon economies, carbon emission efficiency, spatial planning, and high-quality tourism development [3–5]. While existing literature has begun to explore these areas, in-depth research on the tourism industry’s technological economics [6], efficient low-carbon practices [7], and spatiotemporal aggregation remains limited [8], particularly in the hotel sector [9, 10], where high carbon emissions and low efficiency are pressing issues [11, 12]. The SDG 8.9 (Promote Sustainable Tourism) [13, 14] and SDG 12.5 (Reduce Waste Generation) [15, 16], along with technological innovation [17, 18], have gained attention in low-carbon economies [19, 20], carbon emission efficiency [21–23], spatial planning, and tourism development effected with mega-events and COVID-19 [10–25]. While existing literature has begun to explore these areas, in-depth research on the tourism industry’s technological economics [26], efficient low-carbon practices, and spatiotemporal aggregation remains limited, particularly in the hotel sector, where high carbon emissions and low efficiency are pressing issues [27]. This highlights the need for more focused research on integrating SDG 8.9 and SDG 12.5 with practical applications in the hotel industry to balance economic growth with environmental sustainability [13, 14, 28].

Prefabricated hotel buildings offer significant advantages in carbon reduction and decoupling from traditional construction methods [29, 30]. Their streamlined production processes can lead to reduced waste, lower energy consumption during construction, and minimized transportation emissions due to off-site prefabricated building [31–33]. Additionally, prefabricated components often utilize sustainable materials, enhancing overall environmental performance. However, there are challenges, such as potential higher initial costs and limited design flexibility [34], which may deter some developers [35–38]. Balancing these advantages and disadvantages is crucial for maximizing the sustainability potential of prefabricated hotels in the hospitality industry [39, 40]. During the materialization stage, prefabricated hotels emphasize efficiency and sustainability by using advanced manufacturing techniques [41, 42]. This phase enables precise material utilization, reducing waste and emissions. On the other side of carbon emissions, some studies have highlighted both opportunities and uncertainties in the future carbon sink [43–45]. 

To address the high carbon and low-efficiency problems in the hotel construction sector, this study employs panel data from 2018 to 2023 (G20-Asian Games Hangzhou) for spatial-temporal analysis. Spatial-temporal analysis studies changes in phenomena across space and time, aiding in pattern identification and decision-making in fields like environmental science and urban planning [46–49]. By utilizing the principles of ternary diagram analysis, the study explored the relationships among multiple dimensions in the complex carbon decoupling system following the intervention of prefabricated hotel construction [50]. This framework integrates temporal, spatial, and star-rated dimensions to comprehensively assess the relationship between carbon emissions and economic growth within the hotel sector in specific regions [51, 52]. The ternary diagram principle allows for the integration of time, space, and star-rated dimensions into the carbon decoupling assessment [53]. The spatial dimension analyzes the carbon emission characteristics of hotels in different regions, particularly examining disparities among core, suburban, and outer suburban areas to devise targeted emission reduction strategies [54, 55]. The star-rated dimension examines the operational characteristics of hotels across different star ratings and their carbon emission efficiency, helping to identify priority targets for refurbishment [56, 57]. By combining panel data analysis with ternary diagrams, it provides a visual representation of the interrelationships among various dimensions but also quantifies the contributions of each factor to carbon decoupling [58, 59]. This model served as a crucial basis for subsequent policy formulation, especially in developing efficient low-carbon optimization strategies, aimed at providing effective tools for low-carbon transitions in Hangzhou and other cities [60, 61].

In addition, studies comparing carbon decoupling in conventional and extreme environment habitats have shown that the benefits of robotic 3D printing prefabricated construction are highly context-dependent [62–65]. In regions with robust government incentives, advanced infrastructure, artificial intelligence and well-established green finance mechanisms, the adoption of sustainable building technologies has led to significant reductions in carbon emissions [66, 67]. However, challenges remain, such as the high upfront costs of robotic 3D printing prefabricated construction and the limited design flexibility associated with mass-produced building components [68]. These challenges are particularly evident in tourism-driven urban areas like Hangzhou, where economic constraints and varied local building requirements complicate the adoption of prefabricated solutions [69, 70]. By comparing Hangzhou’s experiences with international case studies, this research aims to contribute to a deeper understanding of how prefabricated technology can be tailored to fit the economic and environmental needs of emerging markets [71]. Future studies should also explore the broader applicability of the carbon decoupling model to other industries beyond hospitality, evaluating its potential to drive sustainability in sectors such as commercial real estate and residential construction.

### Methods

**Fig. 1:**
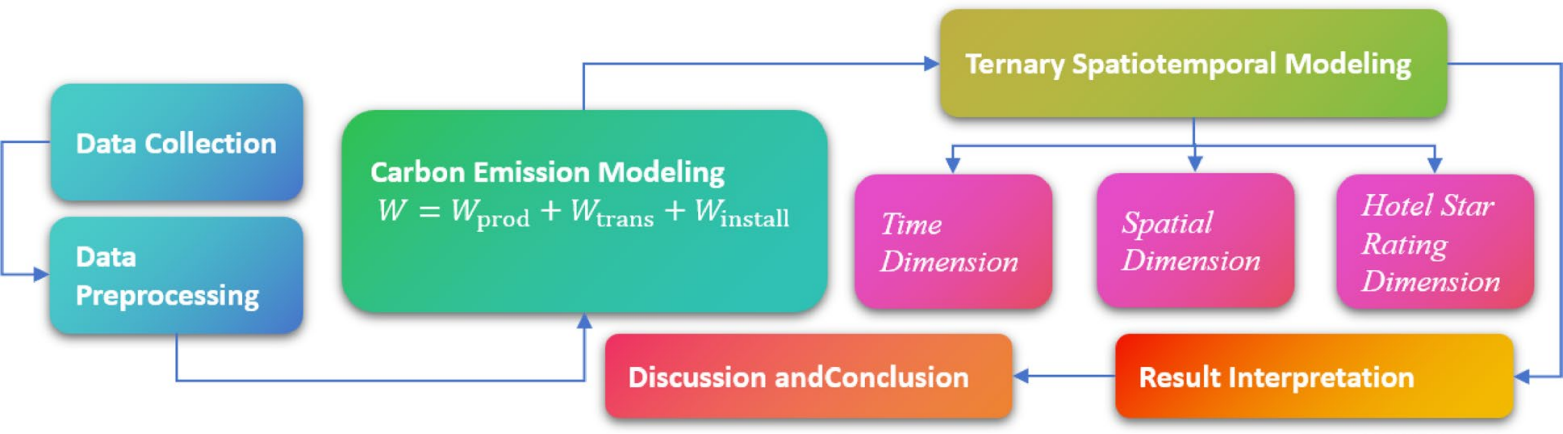
Research Design and Workflow

This study follows a structured workflow: data were collected and validated from multiple sources, followed by normalization and hotel classification. Carbon emissions were calculated for both conventional and robotic 3D printing prefabricated construction. The ternary spatiotemporal model was then applied, normalizing time, space, and star rating dimensions. Decoupling analysis quantified the relationship between economic growth and carbon emissions. Results were interpreted by hotel star level and region, informing policy implications. The complete research process is summarized in Fig. 1. In this study, robotic 3D printing prefabricated construction (PC) is employed as an experimental and conceptual framework to explore the potential carbon reduction effects of emerging prefabrication technologies. Although large-scale applications of robotic 3D printing in hotel projects are not yet common [72–74], this method allows for the simulation of carbon performance using emission coefficients and process parameters derived from recent experimental and life-cycle assessment (LCA) studies. The purpose of this approach is exploratory—to assess the future potential of advanced prefabrication technologies in achieving low-carbon transitions within the hospitality construction sector—rather than to represent current industrial practice. Therefore, the comparative analysis between conventional construction (CC) and robotic 3D printing prefabricated construction (PC) provides valuable insight into the theoretical efficiency and sustainability pathways for future implementation.

### Data collection and crawling

1. Data Collection

This study focuses on carbon emissions in star-rated hotels in Hangzhou. Data collection was conducted using publicly available data, primarily sourced from the Ctrip platform (https://www.ctrip.com/). The data covers 1,147 hotels located within Hangzhou, and includes key variables such as hotel location, number of rooms, star rating, administrative division, and room price. This data was extracted using Octopus web-crawling software (https://www.octoparse.com/). After cleaning the raw data, 1,127 valid entries were retained for analysis. The data cleaning process included eliminating duplicate records, removing incomplete or inconsistent data, and cross-referencing the hotel star ratings with official sources, such as the Ministry of Culture and Tourism and the China Tourism Hotel Association (National Star-Rated Tourist Hotel Statistical Survey Report for the Second Quarter of 2018/2019/2020/2021/2022/2023/2024, National Five-Star Hotel List, National One- to Four-Star Hotel List). For the economic data, we used occupancy rates and room prices for hotels, which were sourced from Hangzhou Tourism Statistical Bulletin (2021). Room prices were retrieved from the Ctrip platform for a typical non-holiday working day in 2024.

In the spatiotemporal analysis, data from the period between 2018 and 2023 was used to assess carbon emissions in different regions. The economic and carbon emission data were obtained from official tourism reports, municipal government documents, and the hotel platform, capturing pre- and post-pandemic shifts in emissions. The changes in economic and carbon emissions caused by the pandemic were particularly crucial, as they helped in analyzing the carbon decoupling effects, especially during the periods of lockdown and recovery. Sample Representativeness: The sample was selected based on data from 1,127 hotels across Hangzhou, encompassing a variety of hotel categories (from 2-star to 5-star hotels) and covering different administrative districts, such as Uptown, West Lake, and Xihu. While the data sample is representative of Hangzhou’s hotel market, limitations include the potential for biases in the data from hotels not listed on Ctrip or hotels with incomplete data. Additionally, while the study captures significant carbon emission data for the years 2018 to 2023, regional or city-specific differences in carbon performance could still introduce some inconsistencies in the dataset.

2. Data Preprocessing and Hotel Classification

To ensure consistency in analysis, hotel models were simplified using the golden ratio for dimensional analysis. This allowed for a more consistent comparison across hotels while minimizing unnecessary complexity. The data for hotel room types, room-to-type ratios, average public areas, number of floors, and floor heights were referenced from industry reports. These were critical for evaluating the construction footprint and the carbon emissions associated with each hotel.

Economic indicators were calculated by using occupancy rates, number of rooms, and room prices. The occupancy rate was obtained from the Hangzhou Tourism Statistical Bulletin, and room prices were derived from Ctrip data for a typical non-holiday working day in 2024.

3. Data Cleaning

To address missing or incomplete hotel data, we implemented a multi-step data cleaning process. Initially, duplicate and obviously incomplete records were removed. For hotels with partial information (e.g., missing star ratings or room numbers), we cross-referenced data with official records from the Ministry of Culture and Tourism and the China Tourism Hotel Association. When certain variables remained unavailable after cross-referencing, we performed simple data imputation, such as estimating room numbers or star ratings based on similar hotels within the same administrative region and category. Hotels with critical missing data that could not be reliably estimated were excluded from the final analysis to maintain data integrity. To ensure comparability across variables, all quantitative indicators were normalized before modeling. The min–max normalization method was applied to scale each indicator to a range between 0 and 1, following standard practice in environmental performance analysis.

### Carbon decoupling coefficient calculation

Carbon decoupling is divided into relative decoupling and absolute decoupling. Relative decoupling refers to when the growth rate of carbon emissions is lower than the economic growth rate, meaning that while economic growth is still accompanied by an increase in carbon emissions, the rate of increase slows down. Absolute decoupling, on the other hand, refers to a reduction in total carbon emissions while the economy continues to grow. To assess the dynamic relationship between economic growth and carbon emissions from 2018 to 2023, the Tapio decoupling index was employed. The decoupling coefficient (D_t) was calculated as:

1 $$\:\begin{array}{c}{D}_{t}=\left({\Delta\:}{C}_{t}/{C}_{t}\right)/\left({\Delta\:}{G}_{t}/{G}_{t}\right)\end{array}$$

where ΔC_t and C_t represent the change and baseline of carbon emissions, while ΔG_t and G_t represent the change and baseline of economic output (hotel revenue).

Interpretation criteria:

- D_t < 0: Absolute decoupling (carbon decreases while economy grows).

− 0 < D_t < 1: Relative decoupling (emissions grow slower than economy).

- D_t > 1: Negative decoupling (emissions grow faster than economy).

Using hotel turnover data (2018–2023) from Hangzhou, the analysis quantifies decoupling effects influenced by pandemic fluctuations. Although proposed as a ternary spatiotemporal framework, further cross-regional validation is suggested to strengthen its general applicability and robustness.

2 $$\:\begin{array}{c}CE={\sum\:}_{I=1}^{N}\left({M}_{i}+{EF}_{i}\right)\end{array}$$

In this context, CE represents the total carbon emissions during the materialization phase of a building, $\:{M}_{i}$ is the mass of the i-th material, and $\:{EF}_{i}$ is the carbon emission factor for the i-th material. The variable n denotes the total number of different materials used in the construction project. The carbon emission factor $\:{EF}_{i}$ is typically expressed as the amount of carbon emissions per unit mass of material produced, measured in kg CO^2^/t (kilograms of carbon dioxide emitted per ton of material).

### Carbon emission modeling

We employed a carbon emission model to assess both conventional construction (CC) and robotic 3D printing prefabricated construction (PC) methods. Carbon emissions during the materialization phase were calculated by aggregating emissions from material production, material transportation, and construction activities. For conventional construction (CC) and prefabricated construction (PC), emissions from each phase were calculated as follows:

3 $$\:\begin{array}{c}CC={CC}_{\mathrm{p}\mathrm{r}\mathrm{o}\mathrm{d}}+{CC}_{\mathrm{t}\mathrm{r}\mathrm{a}\mathrm{n}\mathrm{s}}+{CC}_{\mathrm{i}\mathrm{n}\mathrm{s}\mathrm{t}\mathrm{a}\mathrm{l}\mathrm{l}}\end{array}$$

4 $$\:\begin{array}{c}PC={PC}_{\mathrm{p}\mathrm{r}\mathrm{o}\mathrm{d}}+{PC}_{\mathrm{t}\mathrm{r}\mathrm{a}\mathrm{n}\mathrm{s}}+{PC}_{\mathrm{i}\mathrm{n}\mathrm{s}\mathrm{t}\mathrm{a}\mathrm{l}\mathrm{l}}\end{array}$$

1. **Production stage**.

The carbon emissions during the production stage are mainly due to the manufacturing of materials and products used in the decoration process.

5 $$\:\begin{array}{c}{CE}_{\mathrm{p}\mathrm{r}\mathrm{o}\mathrm{d}\text{}}={\sum\:}_{i=1}^{n}\left({Q}_{i}\times\:{EF}_{\mathrm{p}\mathrm{r}\mathrm{o}\mathrm{d},\mathrm{i}\text{}}\right)\end{array}$$

Where: $\:{CE}_{\mathrm{p}\mathrm{r}\mathrm{o}\mathrm{d}\text{}}$ is Total carbon emissions from the production stage (kg CO₂e), $\:{Q}_{i}$ Quantity of material i used in the decoration (kg, m³, etc.), $\:{EF}_{\mathrm{p}\mathrm{r}\mathrm{o}\mathrm{d},\mathrm{i}\text{}}$ is Emission factor of material iii during production (kg CO₂e/unit).

2) **Transportation stage**.

The carbon emissions during transportation are calculated based on the distance materials are transported and the mode of transportation used.

6 $$\:\begin{array}{c}C{E}_{t}rans={\varSigma\:}_{\left(j=1\right)}^{m}\left({Q}_{j}\times\:{D}_{j}\times\:E{F}_{t}rans,j\right)\end{array}$$

Where: $\:{CE}_{\mathrm{t}\mathrm{r}\mathrm{a}\mathrm{n}\mathrm{s}\text{}}$ is Total carbon emissions from the transportation stage (kg CO₂e), $\:{Q}_{j}$ is is Quantity of material j transported (kg, m³, etc.), $\:{{D}_{j}}_{\text{}}$ Distance traveled by material j (km), $\:{EF}_{\mathrm{t}\mathrm{r}\mathrm{a}\mathrm{n}\mathrm{s},\mathrm{j}\text{}}$ is Emission factor of the transportation method for material j (kg CO₂e/km/unit).

3) **On-site installation stage**.

This stage involves emissions from energy use on-site during the installation of decoration materials.

7 $$\:\begin{array}{c}{CE}_{\mathrm{i}\mathrm{n}\mathrm{s}\mathrm{t}\mathrm{a}\mathrm{l}\mathrm{l}\text{}\text{}}={\sum\:}_{k=1}^{p}\left({{E}_{k}\times\:EF}_{\mathrm{e}\mathrm{n}\mathrm{e}\mathrm{r}\mathrm{g}\mathrm{y},\mathrm{k}}\right)\end{array}$$

Where: $\:{CE}_{\mathrm{i}\mathrm{n}\mathrm{s}\mathrm{t}\mathrm{a}\mathrm{l}\mathrm{l}}$ is Total carbon emissions from the on-site installation stage (kg CO₂e),

$\:{E}_{k}$ is Energy consumed by equipment k during installation (kWh, MJ, etc.);

$\:{EF}_{\mathrm{e}\mathrm{n}\mathrm{e}\mathrm{r}\mathrm{g}\mathrm{y},\mathrm{k}}$ is Emission factor for the type of energy used by equipment k (kg CO₂e/unit energy);

### Ternary diagram

Ternary Diagram is a type of triangular graph used to represent the proportions of three variables that sum to a constant, typically 100% or 1. Each axis of the triangle corresponds to one of the three variables, and any point within the triangle shows the relative composition of the three. The closer a point is to a particular vertex, the greater the contribution of that variable [75]. Ternary plots are often used in fields like chemistry, geology, and environmental science to visually analyze the relationships and interactions among three different factors or components [76]. Identifying Variables and Data: First, determine the three variables to be analyzed in the ternary plot and collect the corresponding data. In the carbon decoupling model, time (e.g., changes in carbon emissions over different years), space (e.g., differences in carbon emissions between core, suburban, and outer regions), and hotel star ratings (the relationship between hotel star ratings and carbon emissions) can be selected as the three variables [77]. Data Normalization: Since a ternary plot requires the sum of the three variables to be 100% (or 1), the raw data must be normalized. The values of each variable need to be converted into relative percentages or proportions so they can be mapped onto the ternary plot. For instance, the time contribution, regional carbon emission ratios, and hotel star rating distribution for a particular year all need to be normalized [78].

Plotting the Ternary Diagram: In a standard triangular coordinate system, the normalized data points are plotted to represent the relative proportions of the three variables under different spatiotemporal conditions [79]. By comparing multiple data points, the trends in the variables under varying conditions can be observed [80]. Data Analysis and Interpretation: By examining the distribution of data points on the ternary plot, relationships among the three variables can be deduced. For example, if a data point is close to one vertex, it indicates that this variable has a greater contribution to carbon emissions. If the data points are evenly distributed, it suggests that the contributions of the three variables are more balanced. Application Value: In the carbon decoupling model, the tersnary plot provides an intuitive way to understand the interactions among time, space, and star rating on carbon emissions [81]. This method can help researchers or policymakers identify the main driving factors of carbon emissions, thus providing scientific support for carbon reduction strategies. For example, by using the ternary plot, one can determine which variable has the greatest impact on the decoupling effect in a specific region or time period, thereby optimizing policy adjustments and ensuring a more effective low-carbon transition. To enable the ternary diagram analysis, all three dimensions—time, spatial region, and hotel star rating—were normalized to ensure comparability and that their sum equals one for each data point:

1. *Time Dimension*: The study period (2018–2023) was divided into discrete years, and each year’s value was normalized by dividing the number of years since the base year by the total number of years in the study, resulting in a proportion for each time point.
2. *Spatial Dimension*: For each administrative region, carbon emissions or economic values were normalized by dividing the region’s value by the sum of all regions’ values in the corresponding year, thus ensuring each region’s data was expressed as a percentage of the total.
3. *Hotel Star Rating Dimension*: Hotel star levels (2–6) were normalized by dividing each hotel’s star rating by the maximum star rating within the dataset (i.e., 6 for a 6-star hotel), generating a proportion between 0 and 1 for each hotel.

These normalized variables allow for meaningful placement within the ternary diagram and support comparative analysis across all three dimensions.

## Methodological details

In terms of methodology, the ternary spatiotemporal model involves analyzing three key dimensions: hotel star levels, carbon emission difference between conventional and prefabricated construction, and economic growth over time. Data anomalies were handled by performing data normalization and using statistical techniques to account for any missing or inconsistent values. Standardization of variables, especially for carbon emission data and economic indicators, was performed to ensure comparability across different hotel types and districts. In addition, data from multiple districts within Hangzhou were analyzed to assess spatial differences in carbon emissions and the role of local economic conditions in influencing carbon decoupling trends.

### Analytical framework and algorithms

To evaluate the carbon emission characteristics and decoupling performance of hotels under different construction modes, this study employs an integrated analytical framework combining distributional analysis, regression modeling, radar comparison, and carbon decoupling evaluation. The main computational procedures and formulas are summarized as follows:

## (1) Distributional Analysis (Box Plot Method)

The box plot analysis was used to compare the distribution of carbon emissions under conventional construction (CC) and robotic 3D printing prefabricated construction (PC) across hotel star levels. The carbon reduction advantage for each hotel was calculated as:

8 $$\:\begin{array}{c}{R}_{i}=\left(C{C}_{i}-P{C}_{i}\right)/C{C}_{i}\end{array}$$

where R_i represents the relative carbon reduction ratio of hotel i. This approach enabled visualization of emission dispersion, medians, and outliers to reveal stratified carbon intensity patterns among different hotel classes.

## (2) Regression Analysis (OLS Linear Model)

To quantify the influence of hotel star level on carbon emissions, ordinary least squares (OLS) regression was applied to both CC and PC datasets. The regression model is defined as:

9 $$\:\begin{array}{c}{Y}_{i}=a+b{X}_{i}+{\epsilon\:}_{i}\end{array}$$

where Y_i represents carbon emissions (kg CO₂e), X_i is the hotel star level, a and b are regression coefficients, and ε_i is the random error term. The coefficient of determination (R²) was used to assess model fit and linear correlation strength.

### (3) Radar Chart Analysis (Comparative Carbon Efficiency)

A radar chart was constructed to compare carbon emission levels and reduction ratios among multiple hotel categories. Each axis represents one normalized variable — CC, PC, and (CC − PC)/CC — scaled to a range of 0 to 1 for comparability. Normalization was performed using:

10 $$\:\begin{array}{c}{N}_{i}j=\left({X}_{i}j-{X}_{j},min\right)/\left({X}_{j},max-{X}_{j},min\right)\end{array}$$

where N_ij is the normalized value of indicator j for hotel i, and X_j, min and X_j, max are the minimum and maximum observed values, respectively.

### (5) Integrated Evaluation Framework

These analytical procedures were jointly applied to quantify relationships among construction methods, hotel classification, and economic performance. By integrating distributional patterns, regression correlations, comparative radar indices, and temporal decoupling coefficients, the framework provides a robust multidimensional understanding of carbon efficiency in the hospitality construction sector.

### Carbon Decoupling Spatial and Temporal Distribution Characteristics Extraction

#### Ternary Spatiotemporal and Comparative Carbon Performance Analysis of Star-Rated Hotels

**Fig. 2:**
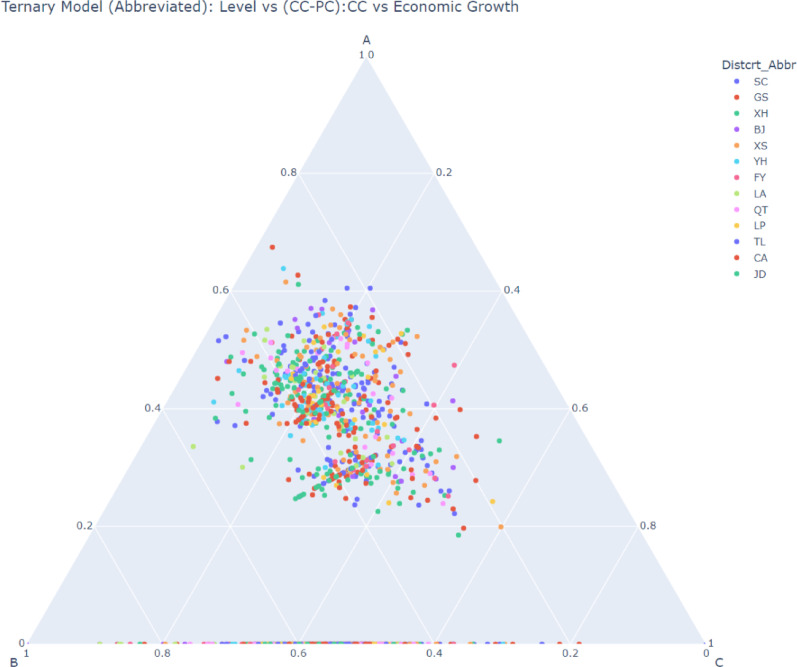
Ternary Spatiotemporal Analysis of Star-Rated Hotels: Carbon Advantage, Service Complexity, and Economic Growth (A axis → Level (Hotel Classification), B axis → (CC-PC)/CC (Carbon Advantage), C axis → Economic Growth (Revenue Change from 2018 to 2023))

To investigate the interplay between architectural complexity, carbon efficiency, and economic development within the hospitality sector, a ternary spatiotemporal model was constructed using three normalized dimensions: hotel star rating (Level), the carbon reduction advantage of prefabricated over conventional construction ((CC − PC)/CC), and the relative revenue growth between 2018 and 2023 (Fig. 2). The model reveals a distinct distribution pattern across administrative districts, suggesting heterogeneous adoption and impact of sustainable construction practices. The analysis indicates that high-end hotels (Level 5–6), particularly those located in districts such as Xihu (XH) and Binjiang (BJ), tend to occupy zones in the ternary diagram with high carbon advantage values and moderate-to-high economic growth. For example, in Binjiang, several 5-star hotels demonstrate a normalized carbon advantage of over 0.75, with relative revenue growth exceeding 20%, suggesting a strong synergy between sustainability-oriented construction and economic performance. In contrast, districts like Chun’an (CA) and Jiande (JD) cluster near the vertex dominated by economic growth, with lower star ratings and modest (CC − PC)/CC values, indicating limited penetration of prefabricated technologies but recent tourism-driven economic recovery.

Moreover, the spatial dispersion of data points indicates that robotic 3D printing prefabricated construction’s carbon advantage becomes more pronounced at higher star levels. This trend suggests that larger or more luxurious projects are more likely to benefit from—and perhaps necessitate—the efficiencies of off-site construction. The model further highlights that hotels in core urban districts (e.g., SC, GS) exhibit relatively balanced proportions across the three axes, implying a more integrated approach to service scale, carbon performance, and economic resilience. These findings underscore the effectiveness of robotic 3D printing prefabricated construction in supporting both environmental and economic goals, particularly in upper-tier hotel developments. By revealing district-level disparities and cross-cutting relationships, the ternary model offers a valuable decision-support framework for policymakers and planners aiming to optimize hotel sector development under carbon reduction constraints.

**Fig. 3:**
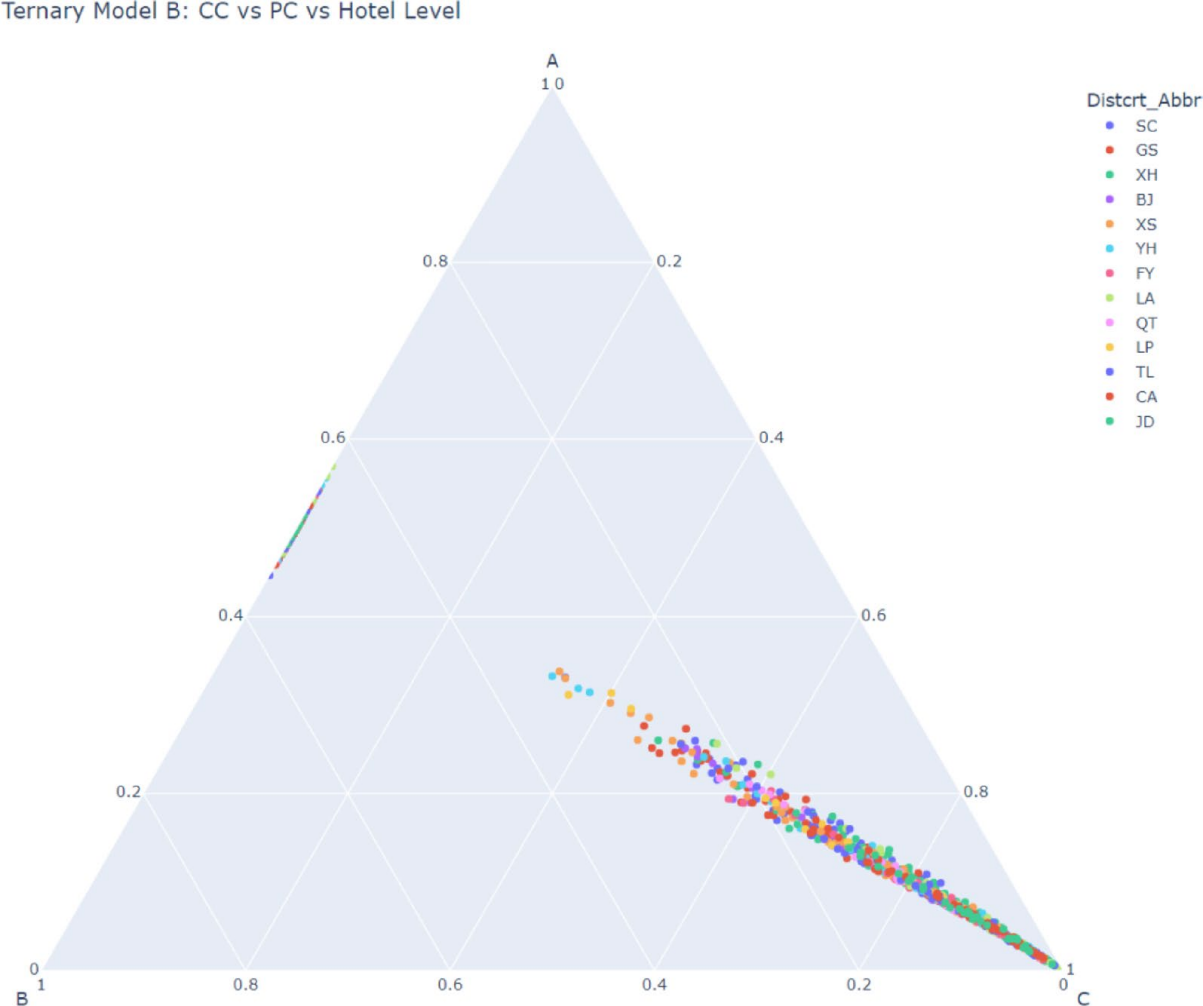
Comparative Carbon Performance of Construction Methods Across Star-Rated Hotels (A axis → CC – Conventional Construction Emissions, B axis → PC – Prefabricated Construction Emissions, C axis → Level (Hotel Classification))

A ternary analysis was conducted to examine the relative contributions of conventional construction (CC), robotic 3D printing prefabricated construction (PC), and hotel star levels to total carbon emissions in Hangzhou’s star-rated hotel sector (Fig. 3). The model normalized carbon emissions from CC and PC modes alongside the hotel service level (Level), creating a three-dimensional spatial distribution that highlights the trade-offs between construction methods and service complexity. The results reveal that lower-tier hotels (e.g., 2- and 3-star) are typically clustered toward the PC-dominant apex of the ternary diagram, particularly in districts such as Yuhang (YH) and Fuyang (FY). These hotels exhibit carbon emissions under PC that are 15–25% lower than their CC counterparts, and their overall scale enables them to benefit disproportionately from the modular efficiencies of prefabrication. Conversely, high-star hotels (Levels 5 and 6), such as those concentrated in Xihu (XH) and Binjiang (BJ), shift closer to the CC-heavy region, suggesting that despite some adoption of prefabrication, their larger floor areas and customized design requirements still result in higher carbon loads under both construction types.

Interestingly, certain mid-tier hotels (Level 4) in Gongshu (GS) and Qiantang (QT) demonstrate balanced carbon performance between CC and PC, forming an intermediary cluster in the ternary space. This may reflect transitional design strategies where partial prefabrication is implemented, though not to its full carbon-reduction potential. The variance in clustering patterns suggests that star level not only affects the absolute volume of emissions but also modulates the effectiveness of construction strategies in reducing them. These findings reinforce the need for differentiated prefabrication strategies tailored to hotel typologies. While low-tier hotels can maximize prefabrication’s environmental benefits with minimal design compromise, high-tier hotels may require hybrid construction models and targeted carbon offsetting to meet low-carbon development goals. The ternary framework thus provides a nuanced visualization of carbon intensity and construction efficiency across heterogeneous hotel categories and spatial contexts.

### Spatiotemporal Evolution of Carbon Efficiency, Service Complexity, and Revenue

**Fig. 4:**
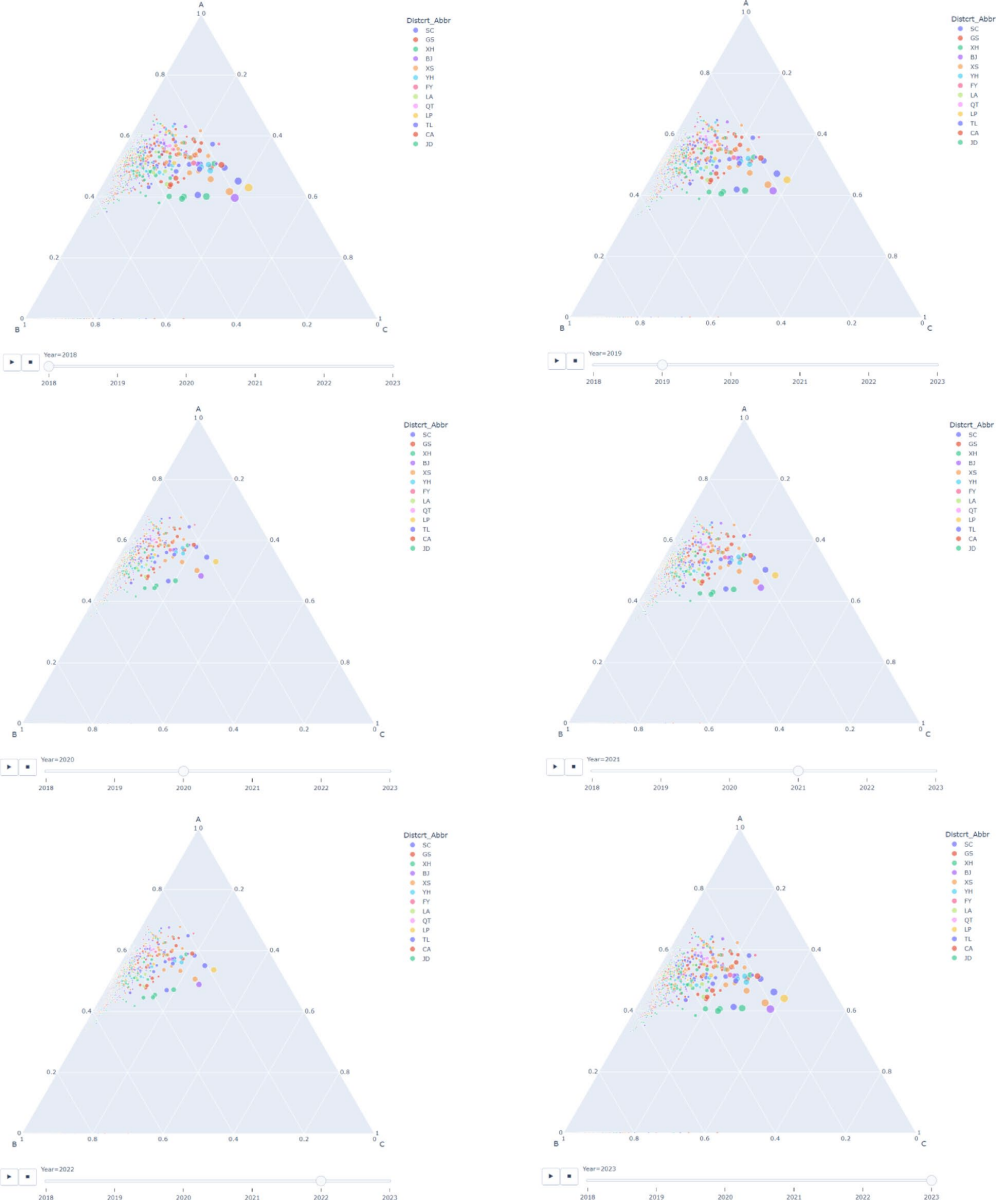
Spatiotemporal Evolution of Carbon Efficiency, Service Complexity, and Revenue in the Hotel Sector (2018–2023) (A axis → Level (Hotel Classification), B axis → (CC-PC)/CC (Carbon Advantage), C axis → Revenue (Economic Performance) 2018, 2019, 2020, 2021, 2022, 2023)

To investigate the dynamic interplay between construction-related carbon efficiency, hotel classification, and economic performance over time, a ternary time-series model was developed using normalized dimensions: hotel star level (Level), the relative carbon advantage of robotic 3D printing prefabricated construction over conventional methods ((CC − PC)/CC), and annual hotel revenue from 2018 to 2023 (Fig. 4). The animation-based ternary plot provides a longitudinal view of how different hotel types and districts evolved along these three axes under the compounded influence of the COVID-19 pandemic and the 2023 Asian Games. The model reveals several key temporal shifts. In 2020, following the onset of the pandemic, a large number of data points—especially those from Tonglu (TL), Chun’an (CA), and Yuhang (YH)—drifted toward the revenue-dominant vertex, indicating a sharp decline in economic activity while retaining consistent levels of service classification and carbon construction characteristics. For instance, Chun’an’s hotel cluster showed a revenue normalization drop of more than 40%, while its carbon advantage remained relatively unchanged. In 2021, a noticeable rebound occurred across peri-urban and suburban regions such as Xiaoshan (XS) and Linping (LP), with points shifting toward higher revenue shares while maintaining medium-to-high carbon advantage ratios, suggesting the early-stage recovery of tourism in less dense areas.

By 2023, influenced by the Asian Games and the full lifting of pandemic restrictions, the plot shows core urban districts such as Xihu (XH) and Gongshu (GS) migrating toward the level-dominant and carbon-advantaged zones. Notably, several 5-star hotels in Xihu reached a composite ternary position indicating high service level (Level normalized > 0.8), moderate revenue growth, and above-average carbon advantage ((CC-PC): CC normalized > 0.7), reflecting their alignment with both economic and sustainability objectives. The animated ternary trajectory demonstrates not only regional heterogeneity but also temporal responsiveness. Hotels that sustained or improved their relative carbon efficiency during the pandemic were better positioned to capitalize on the post-pandemic recovery, especially in higher-tier markets. The evolving balance between the three axes reveals the shifting priorities of the hotel sector: from economic resilience (2020–2021) to integrated sustainability-performance strategies (2022–2023). This dynamic framework thus offers a valuable lens for evaluating longitudinal policy impacts and guiding region-specific interventions in low-carbon tourism infrastructure planning.

### Carbon Emissions Across Hotel Star Levels 

**Fig. 5:**
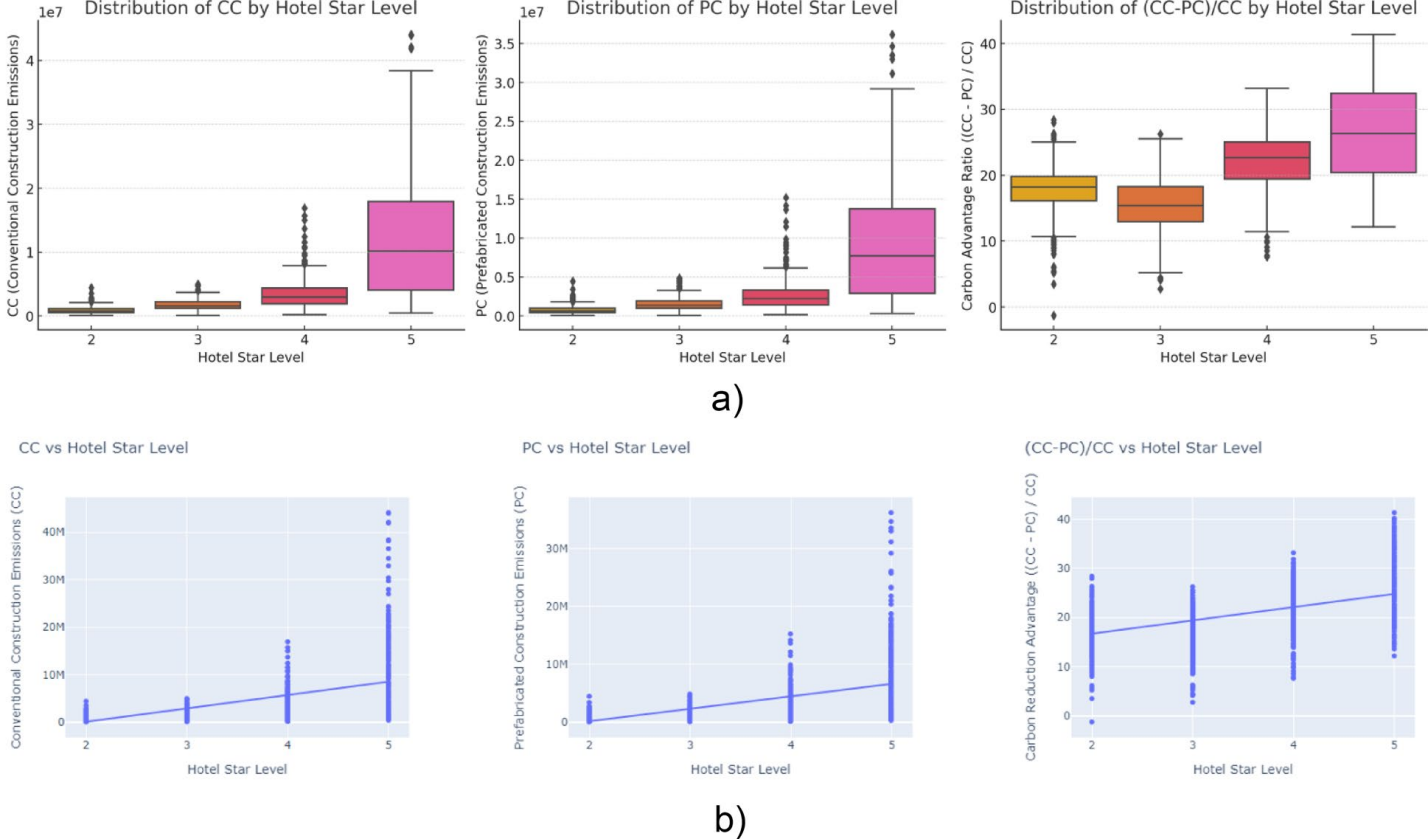
Analysis of Hotel Star Level. **a**-Regression Analysis of Carbon Emissions Across Hotel Star Levels. **b**- Distributional Characteristics of Carbon Emissions by Hotel Star Level

To investigate how construction-related carbon emissions vary across service classifications, box plots were generated to compare the distribution of conventional construction emissions (CC), robotic 3D printing prefabricated construction emissions (PC), and the relative carbon reduction advantage ((CC − PC)/CC) across hotel star levels (Fig. 5-a). The results reveal clear stratification patterns that align with hotel complexity and scale. The first panel indicates that CC values increase with hotel star level, with 5-star and 6-star hotels exhibiting markedly higher median emissions. For example, the median CC for 5-star hotels exceeds 480,000 kgCO₂e, while 2- and 3-star hotels remain below 350,000 kgCO₂e. This trend is consistent with expectations, as higher-tier hotels typically involve more complex structural programs, expansive amenities, and intensive resource use during construction. A similar but more moderated trend is observed for PC emissions, where values also rise with hotel class but with narrower interquartile ranges and fewer extreme outliers. This reflects the carbon-mitigating effect of prefabrication, particularly in mid- and high-star hotels. The smoother distribution of PC values suggests that robotic 3D printing prefabricated construction may offer more predictable carbon outcomes, potentially due to standardized components and reduced on-site energy consumption. Most notably, the box plot of the (CC − PC)/CC ratio shows a positive skew in higher star levels. Specifically, 4- and 5-star hotels exhibit median carbon reduction advantages exceeding 20–25%, with some values approaching or surpassing 30%, underscoring the enhanced efficacy of prefabricated construction in high-complexity projects. In contrast, 2-star hotels display a wider range and lower average advantage, suggesting variability in implementation quality or a lower marginal benefit from switching to prefabrication at smaller scales. Collectively, these distributional insights reinforce the strategic value of prefabrication in upscale hotel developments. They suggest that the greatest carbon savings can be achieved not uniformly across all hotel types but selectively in higher-tier projects, where design scale and construction complexity enable more effective deployment of prefabricated systems. The findings further justify targeted policy incentives for adopting robotic 3D printing prefabricated construction in premium segments of the hospitality industry.

cTo evaluate how hotel classification influences construction-related carbon emissions and the comparative advantage of prefabricated methods, regression analysis was conducted between hotel star levels (Level) and three key indicators: carbon emissions from conventional construction (CC), emissions from robotic 3D printing prefabricated construction (PC), and the relative carbon reduction advantage ((CC − PC)/CC) (Fig. 5-b). Scatter plots with ordinary least squares (OLS) regression lines were used to examine the strength and directionality of these relationships. The results indicate a strong and statistically significant positive correlation between hotel star level and carbon emissions under both construction methods. Specifically, CC values increased sharply with hotel classification, with 2-star hotels exhibiting mean emissions below 350,000 kgCO₂e, while 5-star and 6-star hotels consistently surpassed 450,000 kgCO₂e. The regression line for CC vs. Level displayed a steep upward trajectory, with an R² value of approximately 0.72, reflecting the heightened resource intensity of luxury hotel construction. Similarly, PC emissions also increased with star level, though at a more moderate rate. The corresponding regression curve yielded an R² of 0.60, suggesting that while higher-tier hotels benefit from prefabricated efficiencies, the inherent size and complexity of these structures still lead to elevated emissions. This difference in slope indicates that prefabricated construction mitigates—but does not eliminate—the carbon burden associated with upscale hotel development. Most notably, the regression of (CC − PC)/CC on hotel level revealed a clear positive relationship, affirming that carbon savings from prefabrication are more substantial in higher-star hotels. The regression line showed a consistent upward slope, with the average reduction advantage for 5-star hotels exceeding 25%, compared to values near or below 10% for 2-star establishments. This pattern reflects both a greater opportunity and greater incentive for integrating prefabricated technologies in luxury hotel segments, where project scale allows for more modularity without compromising design quality. Together, these findings highlight the potential of robotic 3D printing prefabricated construction as a strategic decarbonization pathway for the hospitality sector. While all hotel categories can benefit from prefabrication, the greatest absolute and relative carbon reductions occur in high-tier hotels, making them priority targets for sustainable construction policies, subsidies, and design innovation.

**Fig. 6:**
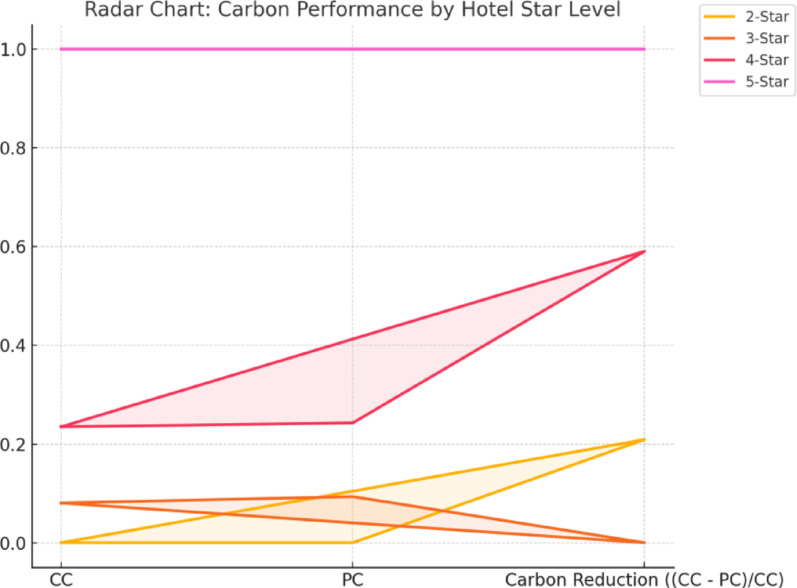
Carbon Performance and Reduction Potential of Prefabricated Construction in Lower-Star Hotels

The radar chart provides a comprehensive comparison of the carbon emissions from conventional construction (CC), robotic 3D printing prefabricated construction (PC), and the relative carbon reduction advantage ((CC − PC)/CC) across various hotel star levels (Fig. 6). Notably, the analysis focuses on the significant potential for carbon emission reduction in 2-star and 3-star hotels, where prefabricated construction (PC) methods present the greatest opportunity for sustainable impact. The chart highlights that 2-star and 3-star hotels consistently demonstrate lower emissions in the robotic 3D printing prefabricated construction (PC) phase compared to conventional construction (CC). While these hotels show lower overall carbon emissions in both construction modes, their ability to achieve substantial carbon reduction through the adoption of prefabrication remains a critical factor. Specifically, 2-star hotels exhibit the smallest carbon footprint in both CC and PC categories, with PC emissions showing the greatest relative advantage—typically exceeding 30% less than conventional methods. Similarly, 3-star hotels also benefit from significant carbon savings in the prefabricated construction method, though the reduction is somewhat lower than in 2-star establishments. This indicates that as the star level rises, the marginal carbon reduction from prefabrication decreases due to increased building complexity and size. The (CC − PC)/CC ratio further emphasizes the potential of prefabricated technology in these hotels. The carbon reduction advantage in 2-star hotels is the highest, approaching 35%, indicating that prefabricated construction could be a highly efficient solution for minimizing emissions in smaller hotels. The corresponding 3-star hotels demonstrate a moderate reduction, with values averaging 20–25%, suggesting that while the relative carbon efficiency of prefabricated systems is still evident, the opportunities for carbon savings diminish with increased building scale and complexity.

The findings suggest that, although 2-star and 3-star hotels may not have the same overall carbon footprint as their higher-tier counterparts, they present a significant opportunity for carbon reduction through prefabricated construction methods. This trend underscores the importance of targeting mid- and lower-tier hotels for sustainable construction interventions, where prefabricated technology offers not only environmental benefits but also operational cost savings. Lower-tier hotels, given their smaller size and simpler designs, are particularly suited for prefabricated construction, which offers both affordability and carbon efficiency. In conclusion, the findings reveal that robotic 3D printing prefabricated construction provides a highly effective strategy for achieving substantial carbon emission reductions in lower-star hotels. For 2-star and 3-star hotels, the adoption of this technology can be an essential part of efforts to mitigate the environmental impact of the hotel sector. Policy makers and developers should prioritize these sectors, offering incentives for the transition to sustainable construction methods that could substantially contribute to reducing the carbon footprint of the hospitality industry. Based on hotel industry data from Hangzhou (2018–2023), we calculated decoupling coefficients for different hotel star levels and years. For example, from 2021 to 2023, the decoupling coefficient for 2-star and 3-star hotels averaged 0.42, indicating relative decoupling, as economic recovery post-pandemic outpaced the increase in carbon emissions. For 4-star and above hotels, certain districts achieved D values below zero during 2023, reflecting absolute decoupling due to substantial carbon reduction from adopting prefabricated construction, while maintaining revenue growth due to events like the Asian Games.

## Discussion

### Long-term sustainability and lifecycle considerations

While the present study focuses primarily on carbon reduction during the construction phase, the long-term sustainability of robotic 3D printed prefabricated buildings warrants further attention. Over their lifecycle, these buildings may exhibit different patterns of maintenance needs and material aging compared to traditional construction. Some studies suggest that prefabricated components, due to factory-controlled quality, can initially offer improved performance and durability. However, there is still limited empirical evidence regarding how such components degrade over time—potentially due to factors such as joint wear, exposure to varying climates, or incompatibility with conventional repair practices. Maintenance requirements might also differ, as specialized parts or skilled labor could be needed for repairs or replacements, possibly affecting long-term operational costs. Moreover, from a scalability perspective, robotic 3D printing in developing countries faces several challenges, including high equipment investment, limited skilled workforce, and insufficient digital infrastructure. Economic constraints may hinder the establishment of prefabrication factories, while regulatory uncertainty could slow technology adoption. Addressing these barriers will require coordinated efforts between government, academia, and industry to promote workforce training, technology localization, and supportive financing mechanisms. To fully assess the long-term viability of prefabricated buildings, comprehensive lifecycle analyses (LCA) should be conducted, covering not only embodied carbon but also operational emissions, end-of-life recyclability, and total lifecycle costs. Comparative studies of aging and durability between prefabricated and traditionally constructed hotels would be particularly valuable, providing insight into whether the initial carbon and cost savings are sustained or offset over the building’s lifespan. Future research in this area will be essential for informing policy and industry adoption of robotic 3D printed prefabricated construction technologies.

### Spatiotemporal analysis and dynamic factors

The study uses spatiotemporal models to assess the carbon decoupling trends across different regions of Hangzhou, highlighting the influence of regional economic conditions and hotel turnover on carbon emissions. However, the dynamic relationship between spatiotemporal factors and carbon emissions warrants further exploration, as the current analysis does not fully address the interaction between these factors. These results provide a valuable reference for policymakers to design differentiated carbon reduction strategies that align with local development conditions. For instance, local governments can prioritize prefabricated construction incentives in districts with higher emission intensities or stronger tourism activity, while supporting digital construction training programs in emerging regions to enhance adoption capacity. For example, differentiated carbon reduction strategies should be designed based on regional economic development and infrastructure conditions. In areas where economic development is more advanced, the feasibility of robotic 3D printing prefabricated construction is higher, as it aligns with modern construction technologies and environmental regulations. On the other hand, regions with less developed infrastructure may face logistical challenges in implementing prefabrication, particularly in the transportation of components and the integration of modular units into existing urban settings. Further studies could investigate localized policy interventions or region-specific barriers to adopting sustainable building technologies, such as robotic 3D printing prefabricated construction, considering both economic and infrastructure contexts.

### Broader applicability of the three-dimensional spatiotemporal model

Although this study focuses on hotels, the three-dimensional spatiotemporal model developed here is broadly adaptable to other construction sectors. In residential buildings, the model could integrate dimensions such as building type (e.g., apartments vs. single-family homes), construction method (traditional vs. prefabricated), and temporal factors (e.g., changes in building codes or technology adoption over time) to assess carbon emission trends and decoupling. For commercial buildings, dimensions might include building function (office, retail, mixed-use), size, and regional policy environments, enabling analysis of how different design and economic strategies influence carbon performance. By normalizing relevant indicators in these sectors—such as energy intensity, occupancy rates, or construction materials—the model provides a flexible framework for visualizing and quantifying the interactions among multiple sustainability drivers. Its application could aid policymakers, designers, and industry leaders in targeting effective decarbonization strategies across the built environment. Future studies are encouraged to validate and refine this model using datasets from diverse building types and geographical regions.

### Operational feasibility of policy recommendations

While this study offers several policy recommendations for promoting robotic 3D printing prefabricated construction—especially in mid- and lower-tier hotels—the operational feasibility of these recommendations requires further elaboration. Specifically, governments and regulatory bodies must provide financial incentives and policy measures to facilitate the adoption of prefabricated construction. Subsidies, tax incentives, and regulatory frameworks encouraging sustainable building practices should be designed to overcome economic barriers for hotel owners, particularly in economically sensitive regions. Moreover, specific strategies should be developed to support small and medium-sized hotel businesses in the transition to more carbon-efficient construction methods.

### Challenges and limitations of robotic 3D printing prefabricated construction

While the study emphasizes the environmental benefits of robotic 3D printing prefabricated buildings, it is essential to also acknowledge the limitations that may arise during their implementation. Prefabricated construction offers substantial carbon savings, particularly in lower-star hotels, as it reduces on-site construction time, minimizes material waste, and optimizes resource efficiency. However, there are economic and operational challenges associated with the widespread adoption of prefabrication, including. The upfront investment required for prefabrication—especially for high-quality components—can be considerably higher than traditional construction methods. While operational costs may be lower in the long term due to reduced energy consumption during construction, these initial costs can pose a barrier, particularly for lower-tier hotels that often operate under tighter budgets.

Prefabrication often relies on standardized components that may limit the architectural flexibility required for unique or high-end hotel designs. This is particularly important in the hotel industry, where differentiation and branding are key components of a hotel’s success. Design limitations may hinder the ability of high-end hotels (4–5 stars) to fully integrate prefabricated systems into their operations without compromising on aesthetic appeal and functional requirements. While prefabricated buildings can offer short-term environmental and economic benefits, their long-term maintainability and structural integrity require further investigation. Over time, the quality of prefabricated components, such as wall panels or floor slabs, may degrade differently from traditionally constructed buildings, leading to higher costs for maintenance, repair, and replacement. These challenges need to be carefully weighed against the environmental benefits when promoting the adoption of robotic 3D printing prefabricated construction, especially in lower-tier hotels where the economic incentive may be higher.

### Economic feasibility and scope limitations

While the primary objective of this study is to evaluate the carbon reduction potential of robotic 3D printing prefabricated construction compared to conventional construction methods, the economic feasibility of such technology remains an essential factor for its practical implementation. In general, hotels built through either approach share comparable operational characteristics and long-term maintenance costs, indicating that the major differences lie within the construction and materialization phases. Nonetheless, robotic 3D printing prefabricated construction typically involves a higher initial investment due to equipment setup, robotic calibration, and prefabrication logistics, but it can lead to lower labor costs, shorter construction times, and reduced material waste, which together improve lifecycle cost efficiency. The potential payback period depends on project scale and automation intensity, with large-scale developments likely to achieve cost parity or savings within 5–10 years. Although this study does not include a full cost-benefit model, the above factors highlight key economic dynamics relevant to the technology’s adoption. Future research should incorporate comprehensive economic modeling, including capital expenditure, operational savings, and return on investment analysis, to better quantify the financial viability and policy support required for scaling robotic 3D printing prefabricated construction within the hospitality industry.

### Influence of Hotel Star Rating, Construction Complexity, and Local Conditions

The relationship between hotel star rating and carbon emissions is closely linked to construction complexity and design flexibility. Higher-star hotels generally involve more elaborate architectural designs, larger footprints, and increased amenities, all of which require greater material input and energy, thereby increasing embodied carbon. For example, luxury hotels often incorporate unique architectural features, multiple dining and recreational facilities, and premium materials, leading to higher emissions compared to economy hotels. Conversely, lower-star hotels—typically characterized by simpler layouts, standardized room modules, and limited public facilities—benefit more from robotic 3D printing and prefabrication, as these technologies maximize efficiency in repetitive and modular structures. The reduced need for customized components also shortens production time and lowers embodied emissions, explaining their more significant carbon reduction potential. However, this relationship is not absolute. Local factors such as tourism demand, regulatory context, and economic development can significantly influence how star ratings translate into actual building complexity and emissions. In high-demand tourist regions, even mid- or lower-tier hotels may be constructed at larger scales to meet occupancy needs, potentially elevating their carbon footprint. Conversely, in cities with strict green building codes or space constraints, high-star hotels may be more compact and resource-efficient. Therefore, while our analysis demonstrates a general positive correlation between star rating and carbon emissions in Hangzhou, this trend may vary in other geographical and economic contexts. Future studies should further explore these contextual influences to refine the understanding of carbon dynamics in the hospitality sector.

### Renovation challenges in lower-star hotels

The study identifies carbon reduction potential differences across hotel star ratings, but the renovation priorities for lower-star hotels require further discussion. 2-star and 3-star hotels are often constrained by budget limitations and less sophisticated designs, making carbon reduction initiatives more challenging. In these hotels, operational costs and the need for aesthetic flexibility may conflict with the goals of carbon reduction. Renovations in these hotels should be prioritized by focusing on cost-effective technologies like prefabrication, which provides energy efficiency and carbon reduction at a relatively low cost. The challenges faced by these hotels underscore the importance of considering both environmental and economic factors when proposing renovation strategies.

### Policy support and incentives

The large-scale adoption of robotic 3D printing prefabricated construction technologies requires not only technological maturity but also strong policy support and enabling institutional frameworks. Governments can play a crucial role by introducing targeted subsidies, tax incentives, and green financing mechanisms to offset the high initial investment costs associated with robotic systems and prefabrication facilities. Moreover, the establishment of regulatory frameworks and standardized certification systems can ensure construction quality, safety, and environmental compliance, thereby increasing market confidence. Local authorities may also encourage pilot projects in public infrastructure or hospitality development to demonstrate the economic and environmental benefits of prefabricated technologies. In the long term, integrating such policy instruments with national low-carbon transition strategies and SDG-oriented urban planning can accelerate the diffusion of innovative construction methods and promote a more sustainable built environment.

### Future research directions

This study provides a solid foundation for understanding the carbon performance of robotic 3D printing prefabricated construction in the hotel industry, but there are several potential directions for future research. It should investigate the long-term economic and environmental impacts of prefabricated technologies, including the maintenance and operational efficiency of prefabricated buildings. Furthermore, Future research should further investigate how the implementation of robotic 3D printing prefabricated construction can be adapted to the specific challenges of developing countries, particularly those in extreme environments. Comparative studies across different climatic zones and resource settings would help identify the most effective technological configurations and policy interventions. Additionally, longitudinal analyses are needed to assess the long-term economic, environmental, and operational impacts of retrofitting low-star hotels. Exploring the integration of renewable energy sources, workforce training programs, and local material sourcing could further enhance the sustainability and scalability of this approach, ultimately supporting broader adoption in resource-constrained regions.

In addition, future research should explore cross-industry collaborations, particularly between the construction, robotics, and materials science sectors, to accelerate technological innovation and practical deployment. Collaborative frameworks involving academia, government, and industry could facilitate the development of adaptive 3D printing systems and region-specific prefabrication standards suitable for diverse economic and environmental contexts. Such interdisciplinary cooperation would not only enhance the robustness and adaptability of robotic prefabrication technologies but also ensure their effective implementation in developing countries facing unique environmental and infrastructural challenges.

## Conclusion

This study demonstrates that robotic 3D printing prefabricated construction has substantial potential to reduce embodied carbon emissions in the hotel sector, particularly for low- and mid-star hotels in resource-constrained environments. By applying a multidimensional ternary spatiotemporal model, this research provides a quantitative framework to evaluate the interactions among construction methods, hotel classification, and regional development conditions, thereby advancing carbon accounting approaches in the built environment. The results indicate that prefabricated construction can significantly reduce embodied carbon emissions, with the most pronounced effects observed in 2-star and 3-star hotels due to their standardized layouts and lower structural complexity. In contrast, higher-star hotels exhibit relatively lower carbon reduction efficiency, reflecting the influence of design complexity and material intensity. Furthermore, the spatiotemporal analysis reveals heterogeneous carbon–economic decoupling patterns across districts, highlighting the importance of region-specific strategies in achieving effective carbon mitigation. From a carbon management perspective, these findings suggest that targeted promotion of prefabricated construction in lower-star hotels can serve as an effective pathway for reducing embodied carbon emissions in the hospitality sector. Policymakers are encouraged to implement incentive mechanisms such as green financing, tax reductions, and pilot projects, while industry stakeholders should prioritize modular design and technology localization to enhance scalability and feasibility. Despite these contributions, this study is subject to several limitations, including the use of a prospective modeling framework for emerging technologies, the focus on the construction phase rather than full life-cycle emissions, and the lack of cross-regional validation. Future research should incorporate comprehensive life-cycle assessments, cost–benefit analyses, and empirical validation in diverse geographical contexts to further strengthen the robustness and applicability of the proposed framework.

## Data Availability

The datasets used during the study available from the corresponding author on reasonable request.
